# Association of sleep apnea and depressive symptoms among US adults: a cross-sectional study

**DOI:** 10.1186/s12889-023-15358-8

**Published:** 2023-03-06

**Authors:** Mei Li, Xue Zou, Hongbin Lu, Fang Li, Yang Xin, Wenwen Zhang, Bo Li, Ying Wang

**Affiliations:** Xi’an International Medical Center Hospital, No.777 Xitai Road, Xi’an, 710100 Shaanxi China

**Keywords:** Sleep apnea, Depressive symptoms, Multivariable logistic regression, NHANES

## Abstract

**Background:**

Sleep apnea exerts adverse health effects due to inflammation and metabolic disruption. It is associated with metabolic diseases. However, the evidence of its relationship with depression is inconsistent. Therefore, this study aimed to investigate the relationship between sleep apnea and depressive symptoms in adults in the United States.

**Methods:**

This study utilized data from the National Health and Nutrition Examination Survey (NHANES), wherein the data from 2005 to 2018 of 9,817 individuals were obtained. Sleep apnea was self-reported by the participants using a questionnaire on sleep disorders. The 9-item Patient Health Questionnaire (PHQ-9) was used to assess depressive symptoms. We implemented multivariable logistic regression and stratified analyses to assess the correlation between sleep apnea and depressive symptoms.

**Results:**

A total of 515 (6.6%) participants among 7,853 non-sleep apnea participants and 269 (13.7%) subjects among 1,964 sleep apnea participants had a depression score ≥ 10, they were deemed to have depressive symptoms. The multivariable regression model, showed that individuals with sleep apnea were 1.36-fold more likely to experience depressive symptoms when adjusted for potential covariates (odds ratios [OR] with 95% confidence intervals of 2.36 [1.71–3.25]), and a positive correlation between depressive symptoms and sleep apnea severity was found. The stratified analyses, revealed that sleep apnea was related to an increased incidence of depressive symptoms in most subgroups, except for those with coronary heart disease. Further, there was no interaction between sleep apnea and the covariates.

**Conclusions:**

Adults with sleep apnea in the US have a relatively high prevalence of depressive symptoms. and the severity of sleep apnea positively correlated with the depressive symptoms.

**Supplementary Information:**

The online version contains supplementary material available at 10.1186/s12889-023-15358-8.

## Background

Depressive disorder is a prevalent psychiatric disorder characterized by high rates of recurrence and disability, which affects approximately 300 million people globally. It is one of the common factors responsible for the burden of disease, and the resulting disease burden is increasing annually. Depressive disorder is projected to become the first disease with a global burden by 2030 [1]. However, to date, there are no biological markers of depression which would make a diagnostic gold standard. Many studies have shown that depressive disorder is often comorbid with a variety of medical conditions [2, 3], which may aggravate depressive symptoms and deteriorate people’s physical health. Therefore, it is imperative to identify and adjust the risk factors to prevent depression.

Sleep apnea is characterized by recurrent pharyngeal airway stenosis and collapse during sleep, which leads to sleep disruption and hypoxemia. The traditional diagnostic method of polysomnography counts the number of breathing obstructions (apneas and hypopneas) during sleep. The apnea hypopnea index (AHI) refers to the number of apnea and hypopnea events occurring per hour of sleep, wherein an AHI of five or more events per hour indicates sleep apnea [4]. Multiple adverse health outcomes have been linked to sleep apnea [5], depression being one of them. Although the relationship between sleep apnea and depression has been explored in several studies, the results remain controversial. Most studies haves indicated that depression is more likely to occur when individuals experience sleep apnea [6–9]. Moreover, a study on pregnant women by Redhead et al. [10] showed that sleep apnea increased the likelihood of depressive symptoms in subjects without a history of mood disorders, and that it aggravated depressive symptoms in subjects with a history of depression. On the other hand, studies have also reported no association [11–14], with one reporting the opposing result of the link of sleep apnea severity a lower incidence of depression [15].

Therefore, we conducted a large-scale study to comprehensively examine the association between sleep apnea and depressive symptoms in adults in the US, based on data from the National Health and Nutrition Examination Survey (NHANES) from 2005 to 2018.

## Methods

### Study population

This cross-sectional study extracted data from 2005 to 2018 from the NHANES database, which is formulated as a complex, multistage, stratified, clustered probability design.. Adult participants (≥ 18 years) who completed the interview and examination at the mobile examination centers (MECs) were selected for our research. We excluded subjects whose nine-item Patient Health Questionnaire (PHQ-9) and questionnaires data on sleep disorders (snoring, gasping, and stopping breathing while sleeping) were missing.

### Description of the variable

#### Outcome ascertainment

Depressive symptom outcomes were screened using the PHQ-9, a screening tool that incorporates the DSM-IV depression diagnostic criteria, which is a proven, reliable and effective resource for both clinical and research settings [16]. The participants answered each item based on the previous two weeks prior to filling the questionnaires, and scored them using the following response categories: 0 (not at all), 1 (a few days), 2 (more than half of the time), and 3 (almost every day), and the total scores ranged from 0 to 27. In our study, the PHQ-9 scores of the subjects were divided into < 10 (without depression) and ≥ 10 (with depression), based on past literature [17].

#### Exposure measurement

Sleep apnea was self-reported by the participants using a questionnaire on sleep disorders (How often did you snore, gasp, or stop breathing while you were sleeping during the past year?) [18]. The classification by frequency of occurrence was as follows: never, rarely (1 to 2 nights per week), occasionally (3 to 4 nights per week), and frequently (5 or more nights per week). Subjects were classified as having sleep apnea with a frequency of rarely, occasionally, or frequently, and the severity of sleep apnea was assessed based on it. The occurrence and severity of sleep apnea were based only on self-reported symptom, and were not backed by polysomnography.

#### Covariate assessment

The covariates were selected based on clinical experience and previous research [5]. Age, sex, race, body mass index (BMI), sleep duration, drink, smoking status, hypertension, diabetes, coronary heart disease(CHD), stroke, total cholesterol, high-density lipoprotein cholesterol (HDL-C), low-density lipoprotein cholesterol (LDL-C), and triglycerides were considered as the potential confounding variables.

The BMI was measured in the MEC and categorized into three groups as: < 25.0 kg/m^2^ (normal), 25.0 to 30.0 kg/m^2^ (overweight) and ≥ 30.0 kg/m^2^ (obesity). As for drinking status [19]: (i) never, in entire life, did not have more than 12 drinks (data from 2005 to 2016) or did not have one drink of any kind of alcohol(data from 2017 to 2018); former, in entire life, had more than 12 drinks (data from 2005 to 2016) or had 0ne drink of any kind of alcohol(data from 2017 to 2018) and during the past 12 months, never drink any type of alcoholic beverage; (ii) mild, less than 2 drinks per day for females, less than 3 drinks per day for males; (iii) moderate, had more than 2 drinks per day for females, more than 3 drinks per day for males; (iv) heavy, had more than 3 drinks per day for females, more than 4 drinks per day for males. Smoking status (never, never smoked, or never smoked more than 100 cigarettes in a lifetime; former, smoked more than 100 cigarettes in a lifetime and currently does not smoke; current, smoked more than 100 cigarettes in a lifetime and currently still smoke). In Tables 1 and 2, we further combined the classifications of smoking and drinking, such as: smoke: Yes (current), No (former, never), Drink: Yes (mild, moderate, heavy), No (never, former). A history of hypertension, diabetes, coronary heart disease or stroke was defined as a self-reported physician diagnosis of hypertension, diabetes, coronary heart disease, or stroke. Cholesterol, HDL-C, LDL-C, and triglycerides were measured in the MEC, and the participants provided samples in the morning after fasting for at least 8.5 h or more but less than 24 h.

**Table 1:** Baseline characteristics of the study participants

Variables	Non-Sleep Apnea	Sleep Apnea	*P*-value
Number of subjects (%)	7853 (79.99)	1964 (20.00)	
Age (years), Mean ± SD	45.7 ± 19.5	49.3 ± 17.4	< 0.001
Sex, n (%)			< 0.001
Male	3687 (47)	1148 (58.5)	
Female	4166 (53)	816 (41.5)	
Race/ethnicity, n (%)			0.223
Mexican American	1522 (19.4)	345 (17.6)	
Other Hispanic	603 (7.7)	162 (8.2)	
Non-Hispanic White	3643 (46.4)	955 (48.6)	
Non-Hispanic Black	1750 (22.3)	419 (21.3)	
Other Race	335 (4.3)	83 (4.2)	
BMI (kg/m^2^), Mean ± SD	28.1 ± 6.4	30.7 ± 7.3	< 0.001
Sleep duration on weekdays (hours), Mean ± SD	7.1 ± 3.3	6.7 ± 2.6	< 0.001
Depression			< 0.001
No	7338 (93.4)	1695 (86.3)	
Yes	515 (6.6)	269 (13.7)	
Smoke, n (%)			< 0.001
Yes	2271 (54.2)	526 (45.9)	
No	1919 (45.8)	621 (54.1)	
Drink, n (%)			0.276
No	2459(35.2)	619 (33.8)	
Yes	4529 (64.8)	1211(66.2)	
Diabetes, n (%)			< 0.001
Yes	727 (9.7)	297 (16)	
No	6782 (90.3)	1560 (84)	
Hypertension, n (%)			< 0.001
Yes	2190 (28.4)	781 (40.6)	
No	5519 (71.6)	1142 (59.4)	
Coronary heart disease, n (%)			< 0.001
Yes	154 (3.2)	79 (6.2)	
No	4656 (96.8)	1187 (93.8)	
Stroke, n (%)			0.016
Yes	164 (3.4)	62 (4.9)	
No	4655 (96.6)	1207 (95.1)	
HDL-C, Median (IQR)	1.3 (1.1, 1.6)	1.2 (1.0, 1.5)	< 0.001
Cholesterol, Median (IQR)	4.9 (4.3, 5.7)	5.0 (4.3, 5.7)	0.337
Triglycerides, Median (IQR)	1.2 (0.8, 1.8)	1.3 (0.9, 2.0)	< 0.001
LDL-C, Median (IQR)	2.8 (2.2, 3.5)	2.9 (2.4, 3.5)	0.025

Data are presented as n (%), median (IQR), or mean (SD)

*BMI* Body mass index, *HDL-C* High-density lipoprotein cholesterol, *LDL-C* Low-density lipoprotein cholesterol, *SD* Standard deviation, *IQR* Interquartile range

**Table 2:** The association between the probability of depressive symptoms and the covariates in the studied group of adults

Variable	OR (95% CI)	*P*-value
**Age**	1.00 (0.99 ~ 1.00)	0.121
**Sex**		
Male	1 (Reference)	
Female	1.70 (1.47 ~ 1.98)	< 0.001
**Race**		
Mexican American	1 (Reference)	
Other Hispanic	1.68 (1.27 ~ 2.21)	< 0.001
Non-Hispanic White	0.91 (0.74 ~ 1.11)	0.349
Non-Hispanic Black	1.17 (0.93 ~ 1.46)	0.175
Other Race	0.89 (0.59 ~ 1.35)	0.588
**BMI**	1.03 (1.02 ~ 1.04)	< 0.001
**Sleep duration**	0.81 (0.77 ~ 0.85)	< 0.001
**sleep Apnea**		
No	1 (Reference)	
Yes	2.26 (1.93 ~ 2.64)	< 0.001
**Smoke**		
Yes	1 (Reference)	
No	1.81 (1.50 ~ 2.19)	< 0.001
**Drink**		
No	1 (Reference)	
Yes	0.83 (0.71 ~ 0.97)	0.017
**Diabetes**		
Yes	1 (Reference)	
No	0.56 (0.46 ~ 0.69)	< 0.001
**Hypertension**		
Yes	1 (Reference)	
No	0.61 (0.53 ~ 0.71)	< 0.001
**Heart disease**		
Yes	1 (Reference)	
No	0.70 (0.46 ~ 1.05)	0.081
**stroke**		
Yes	1 (Reference)	
No	0.48 (0.33 ~ 0.69)	< 0.001
**HDL-C**	0.83 (0.69 ~ 1.00)	0.052
**Cholesterol**	1.03 (0.96 ~ 1.10)	0.399
**Triglycerides**	1.06 (0.99 ~ 1.13)	0.12
**LDL-C**	0.98 (0.87 ~ 1.10)	0.7

*BMI* Body mass index, *HDL-C* High-density lipoprotein cholesterol, *LDL-C* Low-density lipoprotein cholesterol, *OR* Odds ratio, *CI* Confidence interval

### Statistical analysis

As with descriptive analysis, continuous variables were presented as mean ± standard deviation (normally distributed variables), and categorical variables were presented as median (non-normally distributed variables), frequency, and percentage. To compare the differences between the two groups (with or without sleep apnea), chi-square test or t-test was employed, depending on the variables. According to the frequency of sleep apnea, the participants were categorized into three groups: the lowest frequency was the reference group, and tests for linear trends were performed using multivariate regression models.

To assess the relationship between sleep apnea and the risk of depressive symptoms, logistic regression analyses were applied, and odds ratios (ORs) with 95% confidence intervals (95% CI) were calculated. We constructed the prediction model using the data from logistic regression model to check the goodness of fit. Sensitive analysis included stratified analyses and different models in multivariable logistic regression. In the present study, logistic models were adjusted for age, sex, race, BMI, sleep duration, smoking status, drinking, HDL-C, LDL-C, cholesterol, and triglycerides. Previous studies on the clinical characteristics of sleep apnea [20] showed that sleep apnea is associated with an increased risk of diabetes, hypertension, cardiovascular disease, and stroke. A previous study [21] noted that individuals with cardiovascular disease are more likely to experience depression than the general population. Therefore, stratified analyses were conducted to assess the potential modifications of the relationship between sleep apnea and depressive symptoms, including the following variables: gender, BMI, drink, diabetes, hypertension, coronary heart disease, and stroke. Using the clinical cutoff point, we converted a continuous variable into a categorical variable. Multivariate logistic regression was used to assess heterogeneity among subgroups and the likelihood ratio testing was used to examine the interactions between the subgroups and sleep apnea.

The analyses were performed using the statistical software packages R (http://www.R-project.org, The R Foundation) and Free Statistics software version 1.4. Statistical significance was defined as a two-sided *P*-value of < 0.05.

## Results

**Fig. 1:**
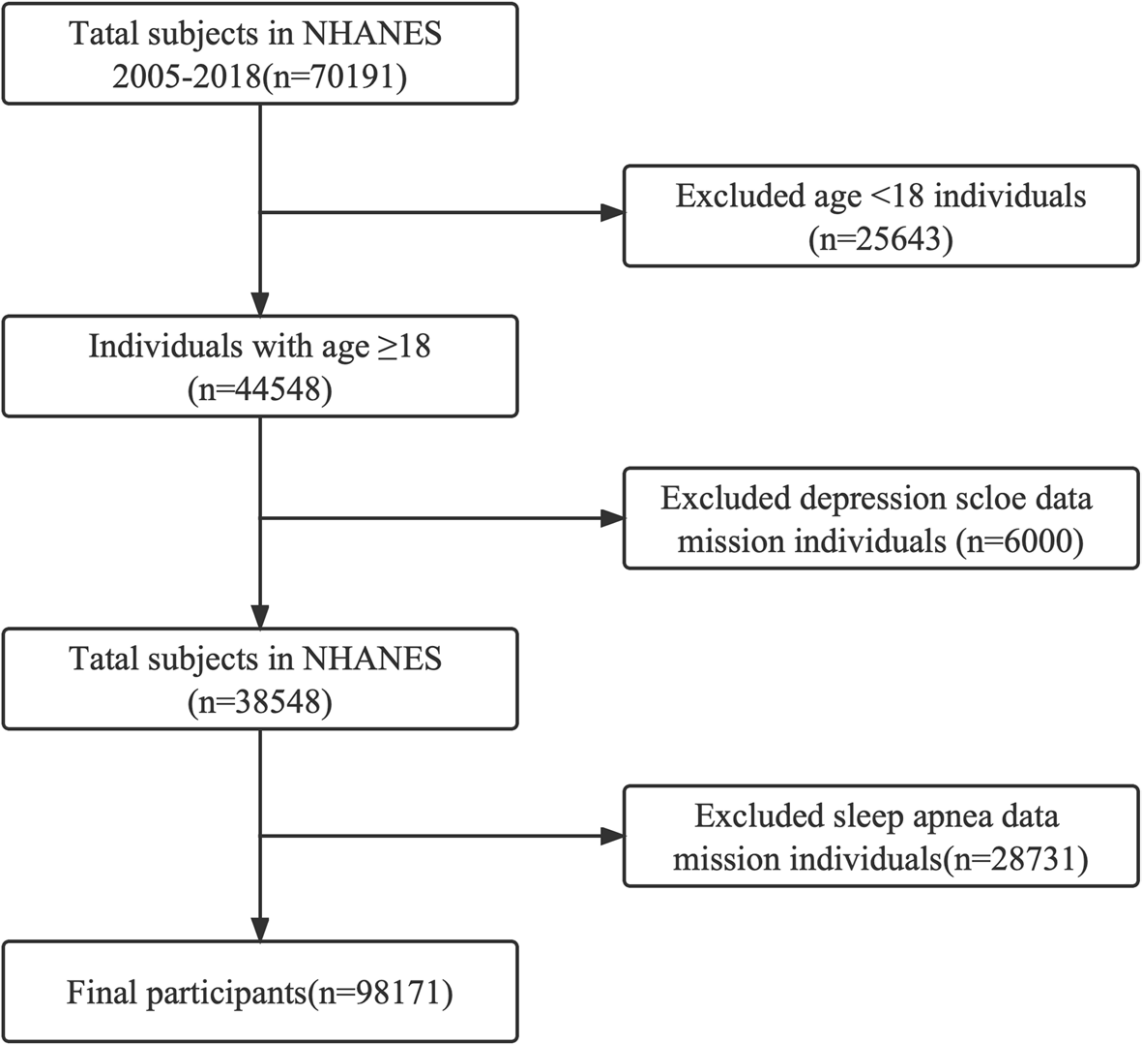
The flow chart of the study. Among 70,191 participants, 25,643 youngers than 18 years of age were excluded. Furthermore, 6,000 participants with missing data on the depression scale score and 28,731 participants with missing data on sleep disorders were excluded. Finally, 9817 participants were included

In this study, we used seven cycles of NHANES, 2005–2006, 2007–2008, 2009–2010, 2011–2012, 2013–2014, 2015–2016, and 2017–2018. A total of 70,191 participants were identified, and 25,643 adults (≥ 18 years old) completed the MEC exam and the interview. Finally, 9,817 participants were included in the present analysis. Participants with missing data on the PHQ-9 (*n* = 6,000) and sleep disorders (*n* = 28,731) were excluded. Finally, 9,817 subjects were enrolled in our study. In Fig. 1, the flowchart for the exclusion criteria can be seen.

Table 1 summarizes the population characteristics of participants with and without sleep apnea. Study participants with sleep apnea were more likely to be older, male, obese, have shorter sleep durations, and drink alcohol than those without sleep apnea. Diabetes, hypertension, coronary heart disease, and stroke were more common in subjects with sleep apnea.

Univariate analysis revealed that sex, BMI, sleep duration, sleep apnea, diabetes, hypertension, and stroke were associated with depressive symptoms (Table 2).

A multivariate logistic regression model with unadjusted and adjusted covariates was used to assess the relationship between sleep apnea with depressive symptoms, and the results are displayed in Table 3. In the unadjusted logistic regression model, individuals with sleep apnea were 1.26-folds more likely to develop depressive symptoms and had an OR (95% CI) of 2.26 (1.93–2.64) compared with individuals without sleep apnea. In Model 1, there was still an association between sleep apnea and depressive symptoms. In Model 2, the ORs (95% CIs) of sleep apnea group subjects were 2.36 (1.71 ~ 3.25). When the sleep apnea subjects were classified by frequency of occurrence, in the unadjusted and adjusted model, the OR (95% CIs) were 2.11 (1.53 ~ 2.9), 2.12 (1.38 ~ 3.26), and 2.03 (1.04 ~ 3.97) for the highest group versus the reference, respectively. As the frequency of sleep apnea increased, the incidence of depressive symptoms significantly increased in both the unadjusted and adjusted models.* P* values for the trend were* P* < 0.001,* P* = 0.001, and *P* = 0.034, respectively.

**Table 3:** Association between sleep apnea and depressive symptoms in multiple logistic regression analyses model

Sleep apnea	n.total	Crude model		Model I		Mode II	
		OR (95% CI)	*P*-value	OR (95% CI)	*P*-value	OR (95% CI)	*P*-value
**No**	7853	1 (Ref)		1 (Ref)		1 (Ref)	
**Yes**	1964	2.26 (1.93 ~ 2.64)	< 0.001	2.20 (1.78 ~ 2.71)	< 0.001	2.36 (1.71 ~ 3.25)	< 0.001
**Severity of sleep apnea**							
**Rarely**	828	1 (Ref)		1 (Ref)		1 (Ref)	
**Occasionally**	592	1.72 (1.25 ~ 2.38)	0.001	1.74 (1.14 ~ 2.67)	0.01	1.91 (1.00 ~ 3.64)	0.05
**Frequently**	544	2.11 (1.53 ~ 2.90)	< 0.001	2.12 (1.38 ~ 3.26)	0.001	2.03 (1.04 ~ 3.97)	0.039
***P-value*** ** for trend**			< 0.001		0.001		0.034

Crude model: No other covariates were adjusted

Model I: adjusted for age, sex, race, body mass index, sleep duration, smoking, and drinking

Model II: adjusted for Model I plus high-density lipoprotein cholesterol, triglycerides, and low-density lipoprotein cholesterol

*OR* Odds ratio, *CI* Confidence interval; Rarely, the frequency of snoring, gasping or stopping breathing while sleeping during the past year was 1 to 2 nights per week; Occasionally, the frequency of snoring, gasping or stopping breathing while sleeping during the past year was 3 to 4 nights per week; Frequently, the frequency of snoring, gasping or stopping breathing while sleeping during the past year was 5 or more nights per week

The result of the goodness of fit was shown in the supplementary table.

**Fig. 2:**
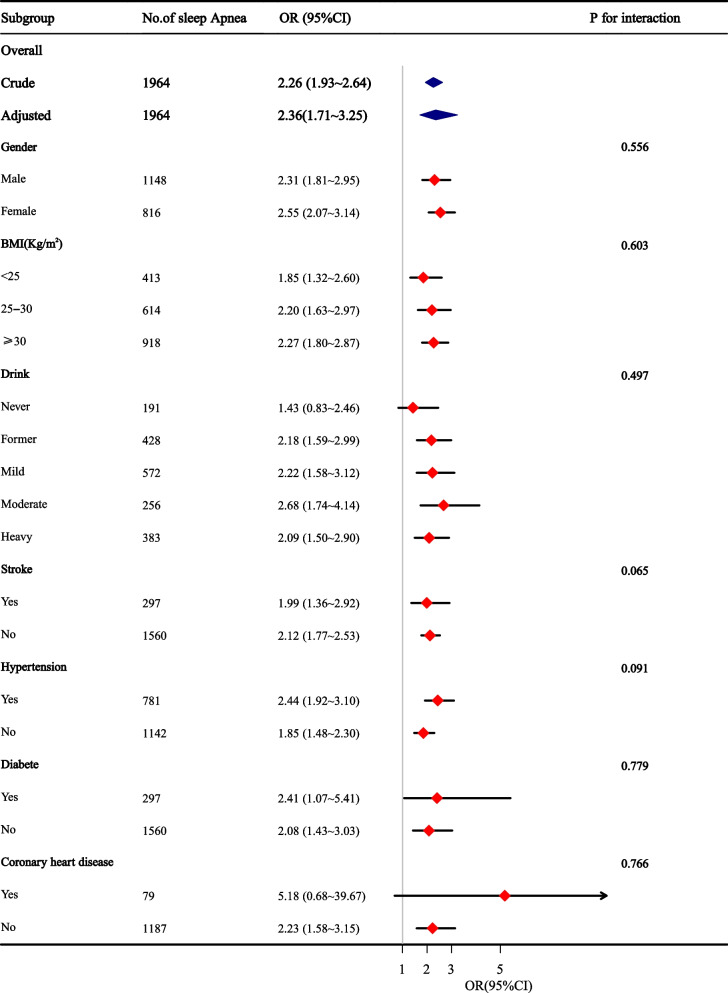
Stratified analyses assessing the effect of sleep apnea on incident depressive symptoms. Results are presented as adjusted ORs (95% CI) of sleep apnea, which were adjusted for age, sex, race, BMI, sleep duration, smoking and drinking, high-density lipoprotein cholesterol, triglyceride, and low-density lipoprotein cholesterol content (except for the variable used for stratification). CI**,** confidence interval, OR, odd ratio, BMI, body mass index

Stratified analyses were conducted to assess the potential modifications of the relationship between sleep apnea and depressive symptoms. As shown in Fig. 2, sleep apnea was related to an increased incidence of depressive symptoms in most subgroups, except for those with CHD. No significant interactions were observed in any subgroups after stratification by gender, BMI, drink, diabetes, hypertension, coronary heart disease, and stroke.

## Discussion

In our study, we discovered sleep apnea to be linked with depressive symptoms among adults in the US. Multivariable analyses adjusted for demographics, BMI, sleep duration, smoking, drinking, and the comorbidities showed that the severity of sleep apnea positively correlated with the occurrence of depressive symptoms, this association was stronger among overweight and obese participants than among other participants. These findings have potential translational implications, in part, because sleep apnea is improvable and treatable, and can have further downstream benefits including improving sleep quality and mood. Therefore, sleep apnea can be targeted in future experimental trials to determine if treatment of sleep apnea influences related mechanisms and disease outcomes.

Nevertheless, the studies reporting conflicting results should be acknowledged [12–14]. A US study [11] indicated that sleep apnea did not appear to be associated with depressive symptoms when assessing mood using the Beck Depression Inventory. Furthermore, a Norwegian study [15] reported that depression, as assessed by the Hospital Anxiety and Depression Scale (HADS), was negatively associated with sleep apnea severity. The reason for the disparities in the outcomes of these studies should be explored with respect to our findings. The use of different scales to assess depressive symptoms may have been responsible for this difference, as for instance, the latter study used a score of eight or higher to indicate depression. A recent meta-analysis [22] evaluated the precision of the depression subscale of the HADS (HADS-D) as a screening tool for depressive symptoms in people with medical conditions and found that, as a tool for screening depressive disorder, the HADS-D cutoff values of 7 or higher were optimal in terms of sensitivity and specificity, while a cutoff value of 8 or higher was less sensitive and more specific. Therefore, to recognize depression in subjects with a medical condition using a cutoff value of 7 with the HADS-D screening tool, would improve the detection rate. Another possibility is that confounding variables is a common drawback of observational research and its impact on our findings could not be fully assessed.

In contrast, the findings of most studies are consistent with our finding [6–8]. A cross-sectional research [10] that investigated the relationship between sleep apnea and depression in pregnant women showed women with sleep apnea to more likely be affected by depressive disorders during late pregnancy. Furthermore, sleep apnea was found to increase depressive symptoms in women who had never been depressed before and exacerbated depressive symptoms in women who had been depressed before. Edwards et al. [23] recruited 426 participants suspected of having sleep apnea from a sleep clinic to examine the prevalence of depressive symptoms among those who had sleep apnea. Similar to our study, their results indicated that depressive symptoms are prevalent in sleep apnea and positively associate with its severity. Although this result is consistent with our findings, most of their study participants were recruited from sleep clinics, which limits the universality of their findings. Therefore, with the large sample size and community-based nature of the present study, we suspect that our study supports this finding with greater strengths.

The relationship and interaction between sleep apnea and depressive symptoms in various subgroups were not examined in other investigations. According to our stratified analyses, patients with both sleep apnea and CHD were more likely to experience depressed symptoms. But this conclusion needs to be interpreted with care due to the small number of people in our study who had both sleep apnea and CHD, and further carefully planned prospective studies in this area are essential.

Although the pathomechanism underlying the relationship between depressive symptoms and sleep apnea remains unclear, several possibilities have been proposed. Individuals with sleep apnea may develop depressive symptoms owing to the disruption of sleep or hypoxemia. Sleep fragmentation or loss leads to excessive daytime sleepiness and adverse moods [24, 25]. Another possible mechanism is neuroinflammation. The pathological process of neuroinflammation is influenced by intermittent hypoxia [26]. An increasing number of recent studies have shown that inflammatory processes’ role in the pathophysiology of depression [27]. Multiple meta-analyses [28–30] have found that, compared to healthy controls, proinflammatory cytokine levels are increased in patients with major depressive disorder. Moreover, anti-inflammatory drugs can ameliorate depressive symptoms [31]. Our study has noteworthy strengths. We utilized several samples from a well-established national investigation in the United States, based on which we performed multiple logistic regression and stratified analyses that resulted in a more accurate and reliable outcome.

Nevertheless, our study has some limitations. Firstly, as this was a cross-sectional study, we could not obtain a result with causality; Secondly, evaluation of sleep apnea was based on self-reporting rather than polysomnography, in the absence of objective indicators. However, snoring, gasping, or stopping breathing while sleeping could most likely be a sign of sleep apnea if the person knows or is told it. Thirdly, the depressive symptoms were measured using the PHQ-9 self-report scale rather than the Diagnostic and Statistical Manual of Mental Disorders, Fifth Edition criteria for major depressive disorders. However, for evaluating depressive symptoms, the PHQ-9 has been proven to have a sensitivity of 0.88 and specificity of 0.80 [32]. The current results should be verified with a of gold standard methods (namely, polysomnography for sleep apnea and DSM-V criteria for depressive disorder) in future research. Finally, our study was to investigate the association between sleep apnea and depressive symptoms in US adults. Though the results were robust, our study couldn’t predict the depressive symptoms, more studies were needed.

## Conclusions

Our study indicated that adults with sleep apnea in the US have a relatively high prevalence of depressive symptoms. The severity of sleep apnea positively correlated with depressive symptoms. Thus, screening for depression is important for the sleep apnea patient population, as further evaluation and treatment for sleep apnea may ameliorate the depressive symptoms and improve the patient’s quality of life.

## Supplementary Information

**Additional file 1: Supplementary Table.** The result of the goodness of fit.

## Data Availability

The datasets analyzed during the current study are available in the National Health and Nutrition Examination Survey repository, https://wwwn.cdc.gov/nchs/nhanes/search/default.aspx.

## Abbreviations

BMI: Body mass index
CI: Confidence interval
HADS: Hospital Anxiety and Depression Scale
HADS-D: The depression subscale of the Hospital Anxiety and Depression Scale
HDL-C: High-density lipoprotein cholesterol
LDL-C: Low-density lipoprotein cholesterol
MEC: Mobile examination center
NHANES: National Health and Nutrition Examination Survey
OR: Odds ratio
PHQ-9: 9-Item Patient Health Questionnaire
