# Supplementary material for: Association of sleep apnea and depressive symptoms among US adults: a cross-sectional study
Source: BMC Public Health. 2023 Mar 6;23:427. doi: 10.1186/s12889-023-15358-8 (PMC9987095; doi:10.1186/s12889-023-15358-8)
Supplement: Supplementary file 1 — Additional file 1: Supplementary Table. The result of the goodness of fit. [file 12889_2023_15358_MOESM1_ESM.docx]

Supplementary Table. The result of the goodness of fit

| Class | Item | Value |
| --- | --- | --- |
| Model likelihood ratio | L.R | 147.085 |
| Rank discrimination indices | C | 0.743 |
| Hosmer-Lemeshow | *P* | 0.132 |
| global Chi-square | $\chi$^2^ | 12.465 |
| discrimination indices | $\mathcal{R}$^2^ | 0.159 |
